# Correction: Advanced care planning during the COVID-19 pandemic: ceiling of care decisions and their implications for observational data

**DOI:** 10.1186/s12904-022-01104-1

**Published:** 2022-12-16

**Authors:** Sam Straw, Melanie McGinlay, Michael Drozd, Thomas A. Slater, Alice Cowley, Stephe Kamalathasan, Nicholas Maxwell, Rory A. Bird, Aaron O. Koshy, Milos Prica, Peysh A. Patel, Samuel D. Relton, John Gierula, Richard M. Cubbon, Mark T. Kearney, Klaus K. Witte

**Affiliations:** 1 Leeds Institute of Cardiovascular and Metabolic Medicine, University of Leeds, Leeds, UK; 2 Leeds Teaching Hospitals NHS Trust, Leeds, UK; 3 School of Medicine, University of Leeds, Leeds, UK; 4 Leeds Institute of Health Sciences, University of Leeds, Leeds, UK

**Correction: BMC Palliat Care 20, 10 (2021)**

**https://doi.org/10.1186/s12904-021-00711-8**

Following the publication of the original article [1], the author reported that there is was an error in Fig. 1 as the blue and red colours are the wrong way around. The correct Fig. 1 is included here and the original article has been updated.

**Fig. 1:**
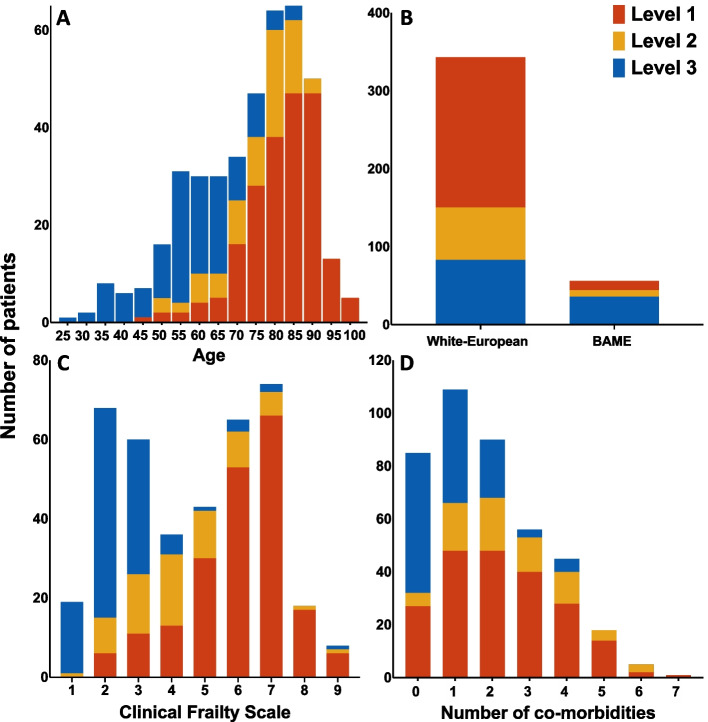
Bar charts showing **a** age, **b** ethnicity, **c** Clinical Frailty Scale and d co-morbidities in patients deemed appropriate for level one, two or three care. Patients in the present study were often elderly, frail and were multi-morbid

## References

[1] Straw S, McGinlay M, Drozd M (2021). Advanced care planning during the COVID-19 pandemic: ceiling of care decisions and their implications for observational data. BMC Palliat Care.

